# Supplementary figures and images for: Pre-treatment subjective sleep quality as a predictive biomarker of tDCS effects in preclinical Alzheimer’s disease patients: Secondary analysis of a randomised clinical trial
Source: PLoS One. 2025 Jan 28;20(1):e0317700. doi: 10.1371/journal.pone.0317700 (PMC11774347; doi:10.1371/journal.pone.0317700)

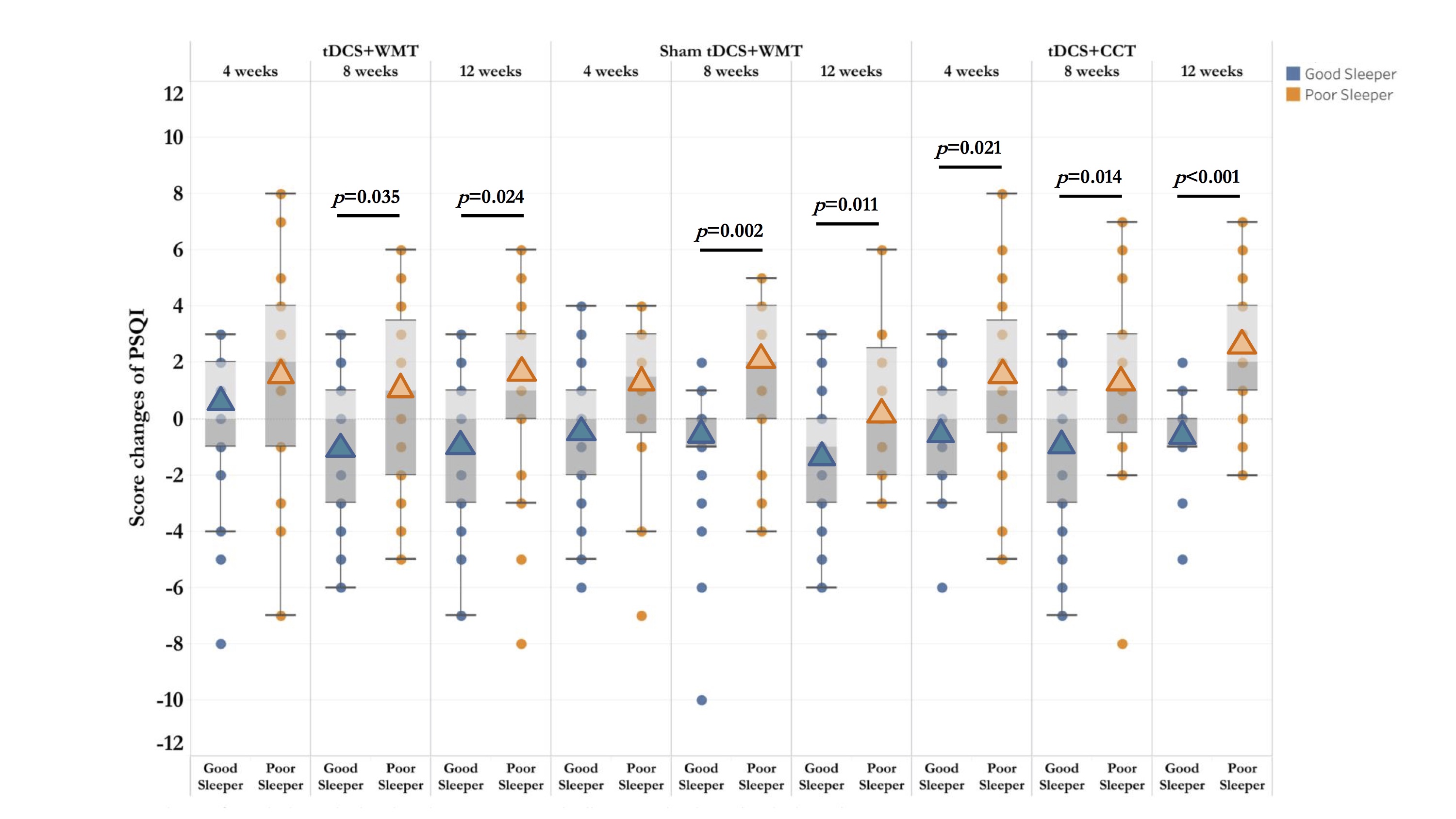

Supplement: S1 Fig — (JPEG) [file pone.0317700.s001.jpg]

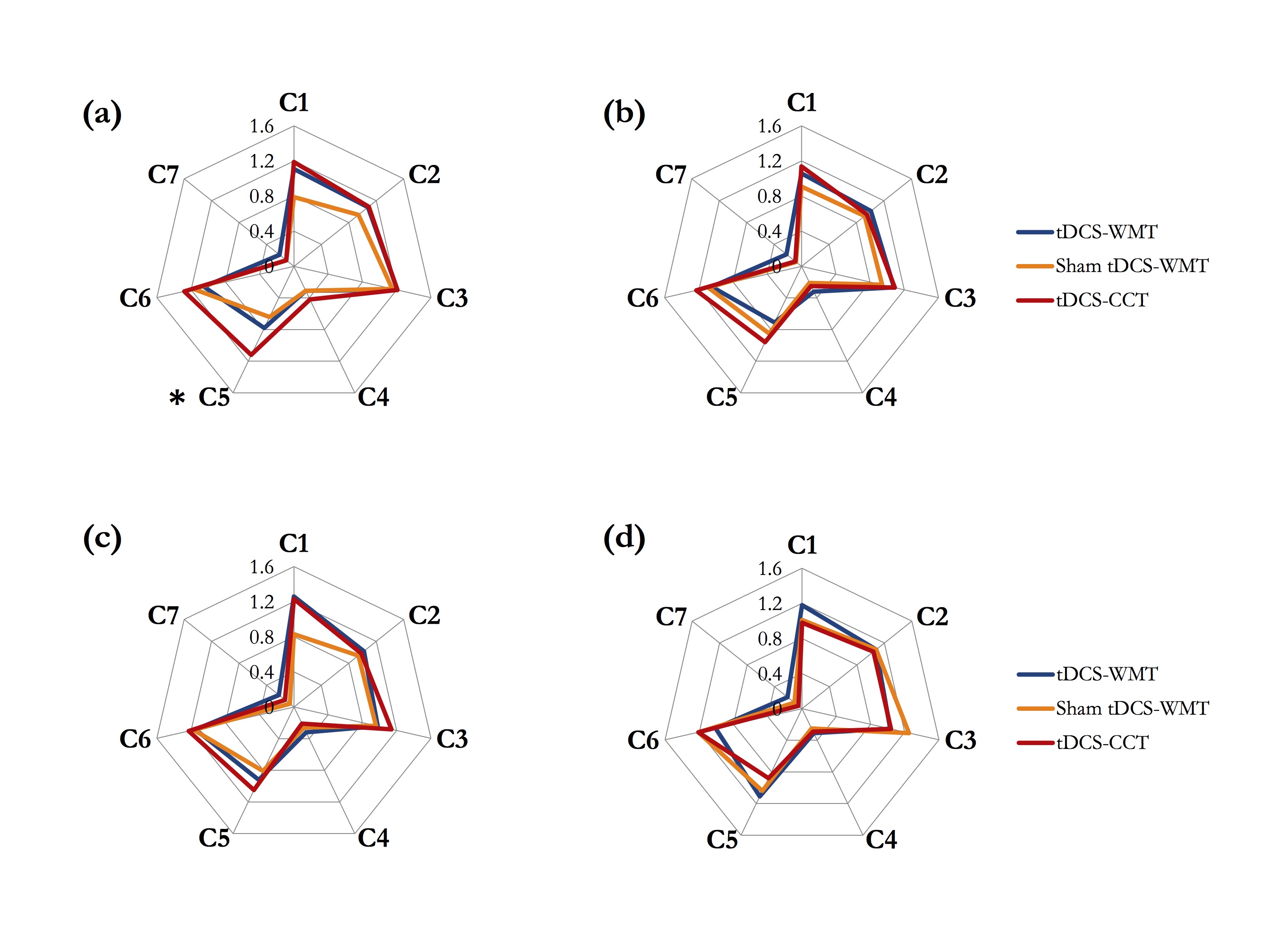

Supplement: S2 Fig — (JPEG) [file pone.0317700.s002.jpg]
