# Supplementary material for: Pre-treatment subjective sleep quality as a predictive biomarker of tDCS effects in preclinical Alzheimer’s disease patients: Secondary analysis of a randomised clinical trial
Source: PLoS One. 2025 Jan 28;20(1):e0317700. doi: 10.1371/journal.pone.0317700 (PMC11774347; doi:10.1371/journal.pone.0317700)
Supplement: S1 Table — (DOCX) [file pone.0317700.s003.docx]

**Supplement Table 1.** Comparations of baseline demographics and global cognition in good and poor sleepers

| Clinical  features | tDCS-WMT (n=62) | | | | Sham tDCS-WMT (n=53) | | | | tDCS-CCT (n=57) | | | |
| --- | --- | --- | --- | --- | --- | --- | --- | --- | --- | --- | --- | --- |
|  | Good sleepers | Poor sleepers | *t* value | *p* value | Good sleepers | Poor sleepers | *t* value | *p* value | Good sleepers | Poor sleepers | *t* value | *p* value |
| Age | 75.72±6.79 | 72.39±5.61 | 2.017 | 0.051 | 74.14±6.68 | 75.21±6.81 | -0.563 | 0.576 | 71.99±5.71 | 73.37±5.99 | -0.857 | 0.395 |
|  |  |  |  |  |  |  |  |  |  |  |  |  |
| Sex (F/M) | 19/16 | 18/12 | -1.104 | 0.211 | 22/11 | 14/6 | -0.247 | 0.806 | 12/10 | 18/17 | 0.225 | 0.822 |
|  |  |  |  |  |  |  |  |  |  |  |  |  |
| Education (years) | 7.84±4.69 | 6.54+4.99 | 1.044 | 0.301 | 6.51±3.66 | 7.05±5.08 | -0.454 | 0.652 | 9.64±4.85 | 6.57±5.32 | 2.188 | 0.033 |
|  |  |  |  |  |  |  |  |  |  |  |  |  |
| ADAS-Cog (total) | 8.75±3.51 | 10.09±4.29 | -1.366 | 0.177 | 9.43±3.33 | 9.11±4.25 | 0.319 | 0.751 | 8.86±3.47 | 10.23±3.62 | -1.407 | 0.165 |
|  |  |  |  |  |  |  |  |  |  |  |  |  |
|  |  |  |  |  |  |  |  |  |  |  |  |  |

Not Note. Data are raw scores and presented as mean (SD).

Abbreviations: tDCS = Transcranial direct current stimulation; WMT = Working memory training; CCT = Controlled cognitive training;

ADAS-Cog = The Alzheimer's Disease Assessment Scale-Cognitive Subscale.
