# Supplementary material for: Pre-treatment subjective sleep quality as a predictive biomarker of tDCS effects in preclinical Alzheimer’s disease patients: Secondary analysis of a randomised clinical trial
Source: PLoS One. 2025 Jan 28;20(1):e0317700. doi: 10.1371/journal.pone.0317700 (PMC11774347; doi:10.1371/journal.pone.0317700)
Supplement: S2 Table — (DOCX) [file pone.0317700.s004.docx]

Supplement Table 2. Treatment outcomes of the components of sleep quality (raw scores)

| Outcomes of  Sleep quality | tDCS-WMT (n=62) | | | | Sham tDCS-WMT (n=53) | | | | tDCS-CCT (n=57) | | | |
| --- | --- | --- | --- | --- | --- | --- | --- | --- | --- | --- | --- | --- |
|  | Baseline | 4^th^ week | 8^th^ week | 12^th^ week | Baseline | 4^th^ week | 8^th^ week | 12^th^ week | Baseline | 4^th^ week | 8^th^ week | 12^th^ week |
| PSQI total score | 5.76±3.66 | 5.43±3.71 | 5.90±3.56 | 5.90±3.81 | 5.11±3.58 | 5.01±3.68 | 5.04±3.43 | 5.92±3.56 | 6.42±3.39 | 5.71±3.51 | 5.96±3.35 | 5.46±3.03 |
|  |  |  |  |  |  |  |  |  |  |  |  |  |
| Component 1 | 1.11±1.06 | 1.06±1.01 | 1.26±1.05 | 1.18±1.14 | 0.79±0.93 | 0.91±1.04 | 0.83±0.95 | 1.01±0.99 | 1.19±0.99 | 1.14±1.02 | 1.23±1.06 | 0.98±0.98 |
|  |  |  |  |  |  |  |  |  |  |  |  |  |
| Component 2 | 1.08±0.52 | 1.01±0.51 | 1.02±0.49 | 1.08±0.53 | 0.94±0.53 | 0.92±0.55 | 0.94±0.49 | 1.08±0.43 | 1.09±0.61 | 0.95±0.55 | 0.98±0.51 | 1.04±0.51 |
|  |  |  |  |  |  |  |  |  |  |  |  |  |
| Component 3 | 1.21±1.02 | 1.08±1.07 | 0.98±1.02 | 1.03±0.99 | 1.15±1.06 | 0.94±0.99 | 0.96±0.98 | 1.25±1.09 | 1.21±0.98 | 1.09±1.07 | 1.14±1.08 | 1.04±1.04 |
|  |  |  |  |  |  |  |  |  |  |  |  |  |
| Component 4 | 0.31±0.61 | 0.32±0.59 | 0.32±0.62 | 0.31±0.56 | 0.31±0.61 | 0.21±0.45 | 0.26±0.63 | 0.25±0.48 | 0.42±0.75 | 0.25±0.51 | 0.21±0.48 | 0.29±0.59 |
|  |  |  |  |  |  |  |  |  |  |  |  |  |
| Component 5 | 0.78±1.01 | 0.71±1.04 | 0.92±1.01 | 1.11±1.19 | 0.64±1.01 | 0.85±1.13 | 0.81±1.11 | 1.04±1.16 | 1.12±1.15 | 0.96±1.14 | 1.05±1.15 | 0.88±1.05 |
|  |  |  |  |  |  |  |  |  |  |  |  |  |
| Component 6 | 1.05±0.79 | 1.05±0.81 | 1.15±0.72 | 1.01±0.66 | 1.17±0.75 | 1.09±0.59 | 1.17±0.64 | 1.21±0.63 | 1.28±0.75 | 1.23±0.76 | 1.23±0.57 | 1.21±0.62 |
|  |  |  |  |  |  |  |  |  |  |  |  |  |
| Component 7 | 0.21±0.69 | 0.22±0.77 | 0.22±0.73 | 0.21±0.71 | 0.11±0.58 | 0.08±0.27 | 0.06±0.31 | 0.11±0.51 | 0.11±0.41 | 0.09±0.39 | 0.13±0.54 | 0.05±0.29 |
|  |  |  |  |  |  |  |  |  |  |  |  |  |

Not Note. Data are raw scores and presented as mean (SD).

Abbreviations: tDCS = Transcranial direct current stimulation; WMT = Working memory training; CCT = Controlled cognitive training; PSQI = Pittsburgh Sleep Quality Index.
